# Use of Ultra-high-frequency Ultrasound on Diagnosis and Management of Lipofibromatous Hamartoma: A Technical Report

**DOI:** 10.7759/cureus.5808

**Published:** 2019-09-30

**Authors:** Daniel Boczar, Antonio J Forte, Luiza P Serrano, Stephen D Trigg, Steven R Clendenen

**Affiliations:** 1 Plastic Surgery, Mayo Clinic Florida - Robert D. and Patricia E. Kern Center for the Science of Health Care Delivery, Jacksonville, USA; 2 Plastic Surgery, University of Florida, Gainesville, USA; 3 Orthopaedics, Mayo Clinic Florida - Robert D. and Patricia E. Kern Center for the Science of Health Care Delivery, Jacksonville, USA; 4 Anesthesiology, Mayo Clinic, Jacksonville, USA

**Keywords:** ultra-high-frequency ultrasound, lipofibromatous hamartoma

## Abstract

Lipofibromatous hamartoma (LFH) is a rare, benign tumor found in the peripheral nerves which is challenging to diagnose. We present a case report of the use of ultra-high-frequency ultrasound (UHFUS) on a patient with an LFH to provide valuable information not available on other imaging modalities regarding tumor invasion of the nerve fascicles.

## Introduction

Lipofibromatous hamartoma (LFH) is a rare, benign peripheral nerve tumor that most commonly affects the median nerve but can also occur in other peripheral nerves [1-3]. Affected individuals may experience swelling of the tissue, and the nerve may increase in length and diameter in the affected area and may be associated with macrodactyly [4]. Nerve compression occurs and can gradually lead to neural symptoms of pain, numbness, paresthesia, and carpal tunnel syndrome. Fibro-fatty strands form in the perineurium and endoneurium, intertwining in the nerves [5]. Therefore, physicians are discouraged to completely excise the tumor due to its invasion within the nerve. Permanent nerve damage is highly likely if the tumor is completely removed by surgery, so treatment for the relief of symptoms is the common practice. Moreover, the macroscopic appearance of the LFH as a yellow mass imposes a challenging differential diagnosis of other nerve tumors, such as neurofibromatosis.

Ultrasound is a non-invasive diagnostic modality that is well-established and used in medicine. It is simple to perform and allows a dynamic image [6-8]. Moreover, it is cost-efficient, being on average 75% less expensive than computed tomography (CT) and 80% less expensive than magnetic resonance imaging (MRI) [9]. However, conventional ultrasound transducers (5 - 20 megahertz (MHz)) are not good at evaluating small structures, such as branches of peripheral nerves [6, 8, 10]. Ultra-high-frequency ultrasound (UHFUS) transducers (frequencies up to 70 MHz) are one of the main improvements that the medical industry has recently made to advance the field of the ultrasonography [7-8, 11-13]. The higher frequency allows a high-resolution image of tiny structures up to 30 μm [6, 8]. UHFUS is a new technology that only a few hospitals have had access to, and few clinical applications have been described in the literature [14-15]. This case report illustrates the UHFUS technology to evaluate a patient with an LFH.

## Technical report

**Figure 1:**
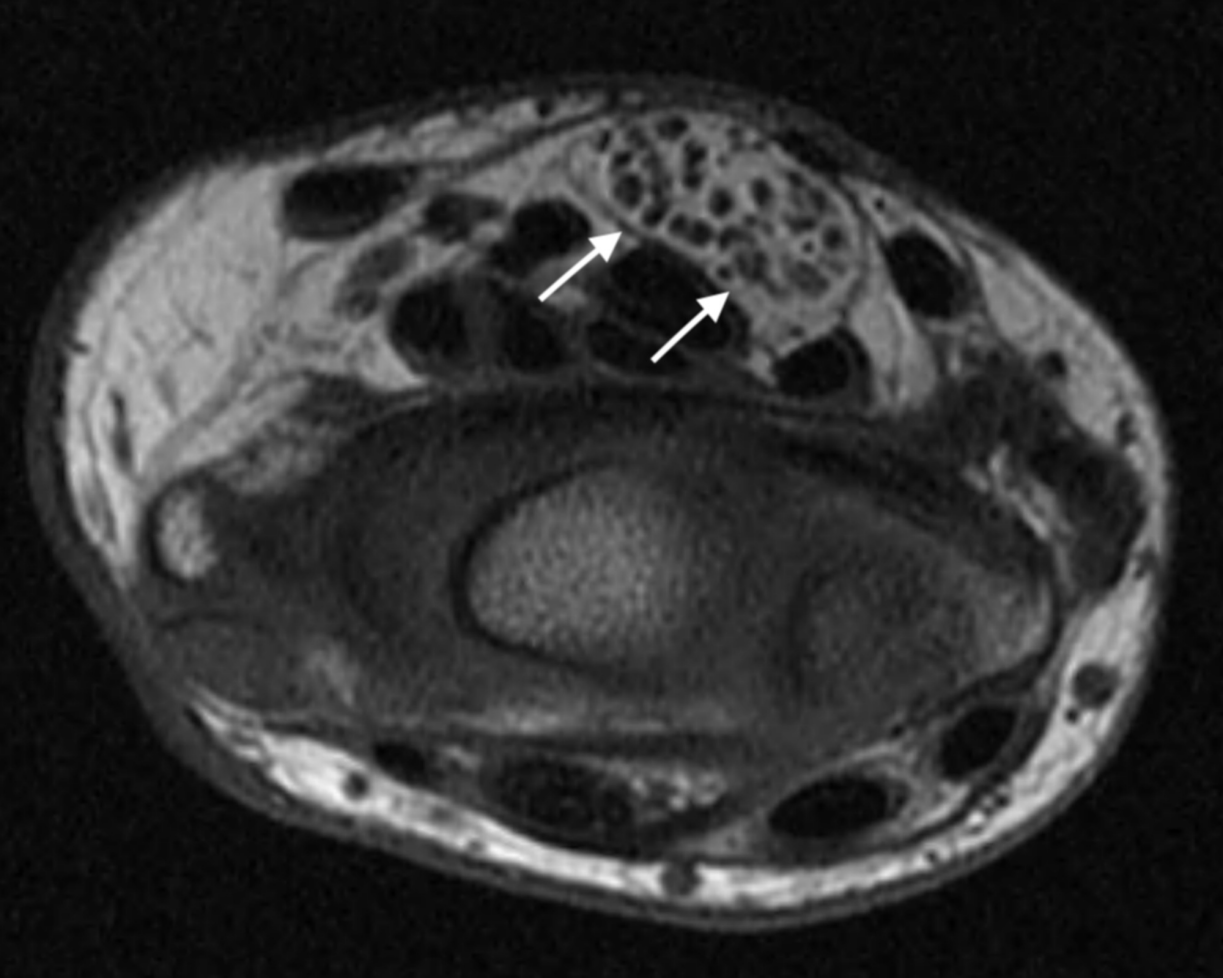
T1 magnetic resonance imaging (MRI) of the left median nerve at the wrist White arrows median nerve

A 27-year-old woman presented complaining about soft tissue swelling on the volar surface of her left wrist that had been present for over six years. She occasionally experienced paresthesia and numbness around the swollen area. The patient complained of progressive sensory disturbance with increasing pain which occurred along the distribution of the median nerve. Both Phalen’s test and Tinel’s sign were positive, indicating carpal tunnel syndrome. The patient underwent exploratory carpal tunnel release surgery with no improvement of her symptoms with biopsy and a histological diagnosis of lipofibromatous hamartoma. The patient then had a T1 MRI which showed common characteristic features correlating with an LFH (Figure 1). 

**Figure 2:**
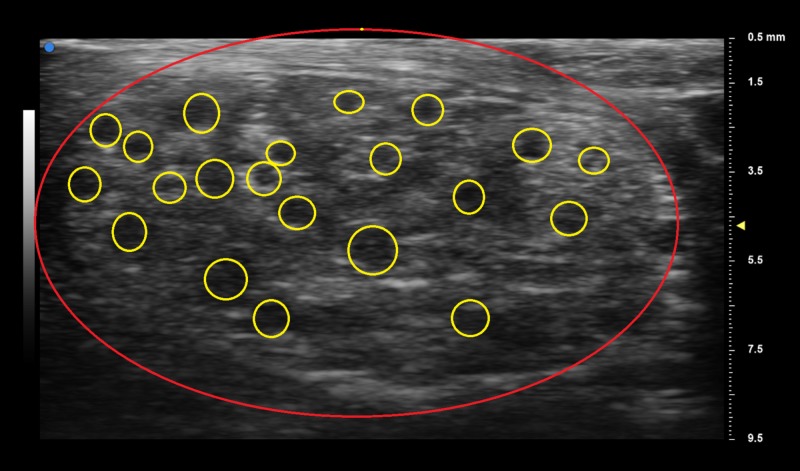
Ultra-high-frequency ultrasound (UHFUS) showing the lipofibromatous hamartoma of the median nerve The red line indicates the circumference of the median nerve; yellow circles surround the nerve fascicles

UHFUS exam was performed on the affected left distal median nerve at the wrist with the Vevo MD ultrasound device (FUJIFILM Visual Sonics, Amsterdam, the Netherlands) using a 70 MHz linear array transducer. Sonography results showed an enlarged cross-section of the median nerve with infiltration of hyperechoic fat separating the nerve fascicles (Figure 2).

**Figure 3:**
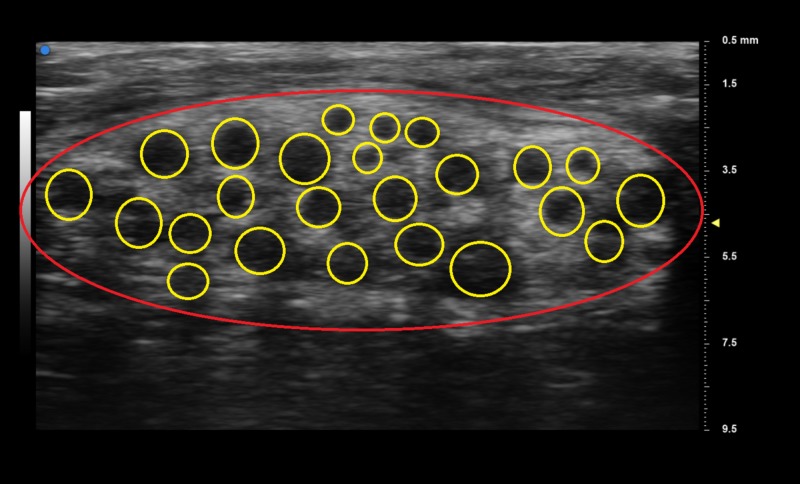
Beginning of the fibro-fatty infiltration of the median nerve in the left forearm The red circle indicates the circumference of the left median nerve; yellow circle surrounds the nerve fascicles.

**Figure 4:**
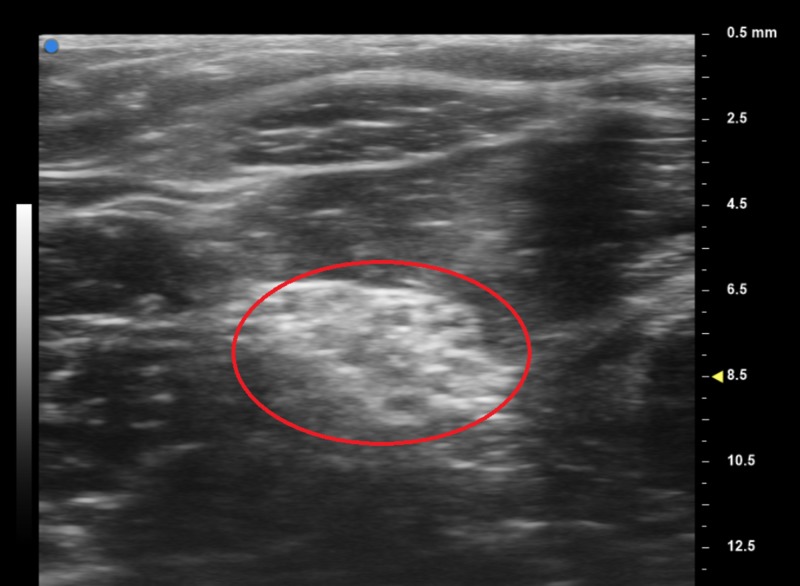
Ultra-high-frequency ultrasound (UHFUS) normal median nerve left forearm The red circle surrounds the left median nerve

Proximally tracing the nerve from the wrist to the forearm demonstrates the beginning of the fibro-fatty infiltration of the median nerve (Figure 3). UHFUS of the patient's left forearm demonstrated a normal-appearing median nerve (Figure 4).

UHFUS of the patient's right wrist shows a normal median nerve (Figure 4).

## Discussion

To our knowledge, this is the first report of UHFUS use in LFH. We demonstrated that UHFUS can be used to assist in identifying an LFH and the extension of the pathology. Moreover, the comparison between images generated by ultrasound on the normal and affected arms showed a potential application of using USFUS to evaluate the progression of the LFH.

Although ultrasound has been used in hand surgery for decades to evaluate carpal tunnel syndrome, the literature about the use of UHFUS to assess peripheral nerve conditions is still in its infancy. Stokvis et al. described the visualization of small cutaneous branches of hand nerves using UHFUS probes [8]. Viviano et al. went further in proposing UFHUS as a diagnostic tool in hand surgery after they conducted a study on one patient and five healthy volunteers. Interestingly, they pointed out that UHFUS can be easily performed by physicians without formal training in ultrasound and without harming the quality of the exam [16]. Schneeberger et al. used UFHUS to monitor graft rejection in a patient with hand allotransplantation [17]. Other applications of UHFUS were described for ophthalmology, cardiovascular, and dermatology [13, 18-20]. 

LHF is difficult to diagnose and a standard of diagnosis has yet to be established; therefore, physicians follow a diagnostic guideline based on their own knowledge and judgment. Symptoms of LFH vary from patient to patient. MRI or sonography is used to diagnose LFH without a necessary need for biopsy. Sonographic results show smooth, rounded, thickened fascicles surrounded by echogenic fatty tissue. MRI results show enlargement of the nerve containing thickened axonal bundles encased in epineural fibrous tissue. The nerve is described as cable-like in the MRI images with a description of an oval appearance [4]. Long cylindrical bands of low T1 and T2-weighted signals are commonly seen.

A differential diagnosis is needed for ganglion cyst, vascular malformation, traumatic neuroma, schwannoma, neurofibroma, and lipoma. LFH is an intraneural tumor, meaning the tumor is found within and around the nerve. It differs from other tumors that are of non-neural sheath origin. Fibro-fatty infiltration around the nerve fascicles pathologically distinguishes LFH, and it has no association with neurofibromatosis. LFH needs radiologic and microscopic investigation for accurate differentiation from neurofibromatosis. Accurate differentiation is essential because neurofibromatosis can progress to malignancy while LFH remains benign. 

Although this study is limited to a case report, we were able to demonstrate the UFHUS capacity to assess nerve fascicles on a patient with LFH. Even though UHFUS provides a high-resolution image of tiny structures, high frequencies imply low pulse length, causing lower tissue penetration and difficulty in the assessment of deep anatomical structures [6, 8, 13]. Therefore, we encourage prospective clinical studies evaluating the use of UHFUS in LFH treatment. 

## Conclusions

We reported the use of UHFUS on a patient with an LFH, in which it provided us information not available through other imaging modalities regarding tumor invasion in the nerve fascicles. We believe this paper could benefit physicians to develop further diagnostic and treatment guidelines for LFH, as well as further research on the use of UHFUS. 
